# Fractionated palliative thoracic radiotherapy in non-small cell lung cancer – futile or worth-while?

**DOI:** 10.1186/s12904-017-0270-4

**Published:** 2018-01-05

**Authors:** Malene Støchkel Frank, Dorte Schou Nørøxe, Lotte Nygård, Gitte Fredberg Persson

**Affiliations:** 1 Department of Oncology, Finsen Center, Rigshospitalet, Blegdamsvej 9, 2100 Copenhagen, Denmark; 2 Section of Radiotherapy, Rigshospitalet, Blegdamsvej 9, 2100 Copenhagen, Denmark

**Keywords:** Non-small-cell lung cancer, NSCLC, Radiotherapy, Palliative, Thoracic, Overall survival, Performance status, Ps

## Abstract

**Background:**

Palliative thoracic radiotherapy (PTR) can relieve symptoms originating from intra-thoracic disease. The optimal timing and fractionation of PTR is unknown. Time to effect is 2 months. The primary aim of this retrospective study was to investigate survival after PTR, hypothesizing that a significant number of patients received futile fractionated PTR. The secondary aim was to find prognostic factors to guide treatment decisions.

**Methods:**

Patients with non-small-cell lung cancer (NSCLC) planned for PTR in the period of 2010-2011 at the University Hospital of Copenhagen were included. We noted pathology, tumor, node and metastasis (TNM) classification of malignant tumors, stage, indication, start date, schedule for PTR, completed y/n, performance status (PS) and time of death. Analyses were performed as an intention-to-treat using Cox regression, Fishers exact test and Kaplan Meier.

**Results:**

A total of 159 patients were included. Median overall survival (OS) was 4.2 months. Sixteen patients (10%) did either not begin or finish PTR. Of these, eight (5%) died prior to or during PTR. Of the 151 patients receiving PTR, sixteen patients (11%) died within 14 days, thirty-three (22%) within 30 days and fifty (33%) within 2 months. PS 0-1 and squamous cell carcinoma were correlated with a better survival.

**Conclusions:**

Our study show that a significant number of patients who received PTR died before they could achieve optimal effect of the treatment. PS and histology were significant prognostic factors favoring PS 0-1 and squamous cell carcinoma. Based on our study, we suggest that patients with PS 0-1 should be considered for fractionated PTR whereas patients with PS ≥ 2 should be considered for high dose single fraction only or supportive palliative care.

**Electronic supplementary material:**

The online version of this article (10.1186/s12904-017-0270-4) contains supplementary material, which is available to authorized users.

## Background

Non-small-cell lung cancer (NSCLC) is the leading cause of cancer related death [1]. When diagnosed, more than 50% of the patients have distant metastases. 40% of the patients have signs or symptoms originating from the thorax with dyspnoea, cough, haemoptysis, recurrent pneumonia or chest pain [2].

Median overall survival (OS) (all stages) without treatment or with platinum-based chemotherapy is 7 months and 8-10 months, respectively [3]. Considering the poor prognostic setting and the clinical symptoms which often affect quality of life (QoL), it is important to focus on a meaningful palliative strategy.

Palliative thoracic radiotherapy (PTR) can relieve symptoms originating from intra-thoracic malignancy and improves QoL in approximately one third of all patients [4].

An optimal radiotherapy regimen will palliate symptoms with minimal toxicity and consider the time investment for the patient. A recent systematic review [5] found no consistent evidence that longer, more fractionated regimens gave better or more durable palliation. Furthermore, there was no significant survival advantage associated with longer regimens with higher biological doses.

The American Society for Radiation Oncology’s (ASTRO) evidence-based clinical practice guidelines suggest that patients with good performance status (PS) may benefit from a higher-dose/fractionated regimen (30Gy/10F equivalent or greater) in terms of a modest survival benefit. Various shorter fractionated schedules (e.g. 20Gy/5F, 17Gy/2F and 10Gy/1F) provide symptomatic relief and can be used for patients requesting shorter total treatment courses or who have a poor PS [6]. Another important factor when choosing palliative strategy is time to effect. In a randomized study comparing 30Gy/10F to 16Gy/2F, the median time to effect was 7 weeks and 5 weeks respectively [7]. An individualized strategy based upon PS, symptom severity, the choice of the patient and an estimation of life expectancy has proven important when choosing a fractionated schedule and whether to give PTR or not [8–10]. Supportive care alone should be considered. Walasek et al. [11] showed that patients in a poor PS (3-4) and stage IIIB/IV experienced equally symptom palliation and survival with radiotherapy compared to supportive care alone. PS is considered the predominant prognostic factor and a significant predictor for futile radiotherapy at the end of life [8, 9, 12–20].

We hypothesize that a significant part of fractionated PTR to patients with NSCLC is futile as patients do not live long enough to achieve the complete effect of the treatment. In this article, we will use the word futile as being ineffective or insufficient and will argue that PTR administered within the last 30 days of life is futile.

In this retrospective study, we investigated OS after PTR and aimed to find prognostic factors to guide treatment decisions.

## Methods

Patients planned for PTR between the 1st of January 2010 and 31st of December 2011 at the University Hospital of Copenhagen (Rigshospitalet) were identified in our ARIA database (Varian medical systems). The standard palliative radiotherapy schedules were 30Gy/10F, 25Gy/5F, 15Gy/3F and 10Gy/1F. Only patients with pathologically confirmed NSCLC were included for further analysis. Date of birth, date of diagnosis, pathology including epidermal growth factor receptor (EGFR)-status (if available), tumor, node and metastasis (TNM) classification of solid, malignant tumors (7th edition), stage, treatment schedule, PS at time of subscription of PTR, indication for PTR and time of death was extracted from the electronic patient chart. If missing, PS was estimated based on chart notes. Fractionated schedule, date of radiotherapy (start/end) and number of fractions received were retrieved through the ARIA planning programme. Prescription date was set 2 weeks prior to PTR. In cases where patients died prior to or did not begin PTR, the actual prescription date was noted. Data lock was 5th of April 2016. PTR was considered futile if patients died within 30 days of treatment start.

### Statistical analysis

Statistical analyses were based on intention to treat (from prescription time to death). Kaplan-Meier was used for survival analysis and cox regression was performed for the multivariate analyses. We investigated PS, pathology, stage, fractionated schedules (30Gy/10F versus 25Gy/5F) and age below median age of the population (70 years). A two-sided *p*-value below 0.05 was considered statistical significant.

## Results

A total of 216 patients were referred for PTR between the 1st of January 2010 and 31st of December 2011. Fifty-two patients had a diagnosis other than NSCLC. Five patients without follow-up were excluded. A total of 159 patients were included for further statistical analysis. Primary treatment was first treatment given to the patient after diagnosis.

**Fig. 1:**
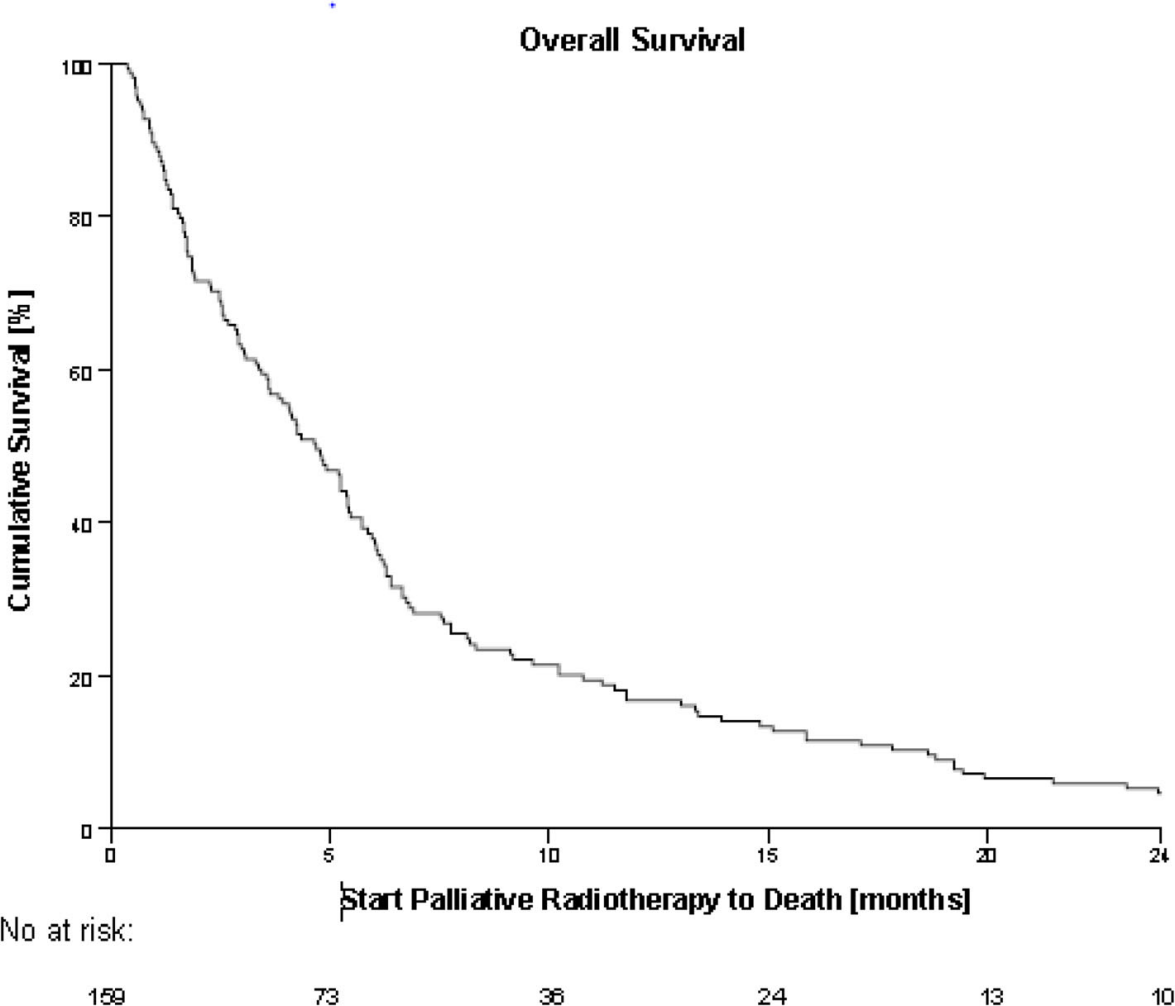
Kaplan-Meier curve showing survival in all 159 patients referred to PTR. Median OS from prescription date of PTR to death = 4.2 months

Patient-characteristics are shown in Table 1. We found that PS was missing in approximately one third of the patients. The group was heavily pretreated before PTR with 18 patients (11%) received >3 regimes of chemotherapy. We did not note if a patient received chemotherapy after PTR but PTR was in almost all cases the last treatment that the patient received. This can be seen in the median OS of 4.2 months from time of prescription of PTR to death. See Fig. 1. A relative large group of patients had stage I-II disease and could potentially be candidates for curative radiotherapy together with a subset of patients with stage III disease. This treatment was not given due to comorbidity and/or PS not suitable for curative radiotherapy.

**Table 1:** Patient-characteristics (*n* = 159)

Patient Characteristics	no (%)
Age at PTR start (years)	
< 70 years	78 (49)
≥ 70 years	81 (51)
Histology	
Adenocarcinoma	77 (48)
Squamous cell carcinoma	69 (43)
Mixed adeno-squamous cell carcinoma	11 (7)
NA	2 (1)
Stage	
I	5 (3)
II	7 (4)
III	53 (33)
IV	94 (59)
WHO Performance Status	
0	14 (9)
1	62 (39)
2	47 (30)
3	33 (21)
4	3 (2)
Indication for PTR	
Dyspnea	58 (30)
Pain	47 (24)
Hemoptysis	22 (11)
Cough	18 (9)
Vena cava superior syndrome	16 (8)
Dysphagia	4 (2)
Other	30 (15)
PTR Schedules	
30Gy/10F	101 (64)
25Gy/5F	50 (31)
15Gy/3F	7 (4)
10Gy/1F	1 (1)
Primary Treatment after diagnosis	
Chemotherapy	74 (47)
Radiotherapy	58 (36)
Surgery	12 (8)
Other	8 (5)
Not treated	3 (2)
NA	4 (3)
Number of Chemotherapy Regimens	
0	59 (37)
1	35 (22)
2	26 (16)
3	16 (10)
> 3	18 (11)
NA	5(3)

Sixteen patients (10%) did not complete PTR: Eight (5%) died prior to or during PTR, four patients were in too poor a condition, two patients withdrew their consent, one had too large a target and for one patient the reason was not stated. Of the 151 patients receiving PTR, sixteen patients (11%) died within 14 days. Thirty-three patients (22%) died within 30 days and fifty patients (33%) died within 2 months of treatment.

Dyspnea and pain were the most frequent stated indications for PTR. Each patient could be noted for more than one indication. The most frequent fractionated schedule was 30Gy/10F (64%).

The median time from prescription to death of all patients was 4.2 months (0-73). Two patients were still alive at data lock (Fig. 1)*.*

Overall, the most common fractionated schedule was 30Gy/10F and thereafter 25Gy/5F. Most patients dying within 60 days of treatment received 25Gy/5F followed by 30Gy/10F and only three patients (3%) received a shorter fractionation. The difference between the regimens was based on PS as our guidelines suggested a shorter fractionation to patients in PS > 2. Haemoptysis was present in 11% of the patient-cases but only 4% received 15Gy/3F, which was the recommended fractionated schedule for haemoptysis. As EGFR-status was not routinely performed at our institution in 2010-2011 but only a selected group of patients, e.g. young women and/or never smokers, we only obtained EGFR-mutational status in 13 patients of which one had mutation in exon 19. These numbers were too small to analyse.

Only PS and histology were statistical significant in cox univariate analyses and were included for further analysis. Cox regression analysis for stage, fractionated schedules and age below the median can be found in (Additional file 2: Figure S2 and Additional file 3: Figure S3)*.*

**Fig. 2:**
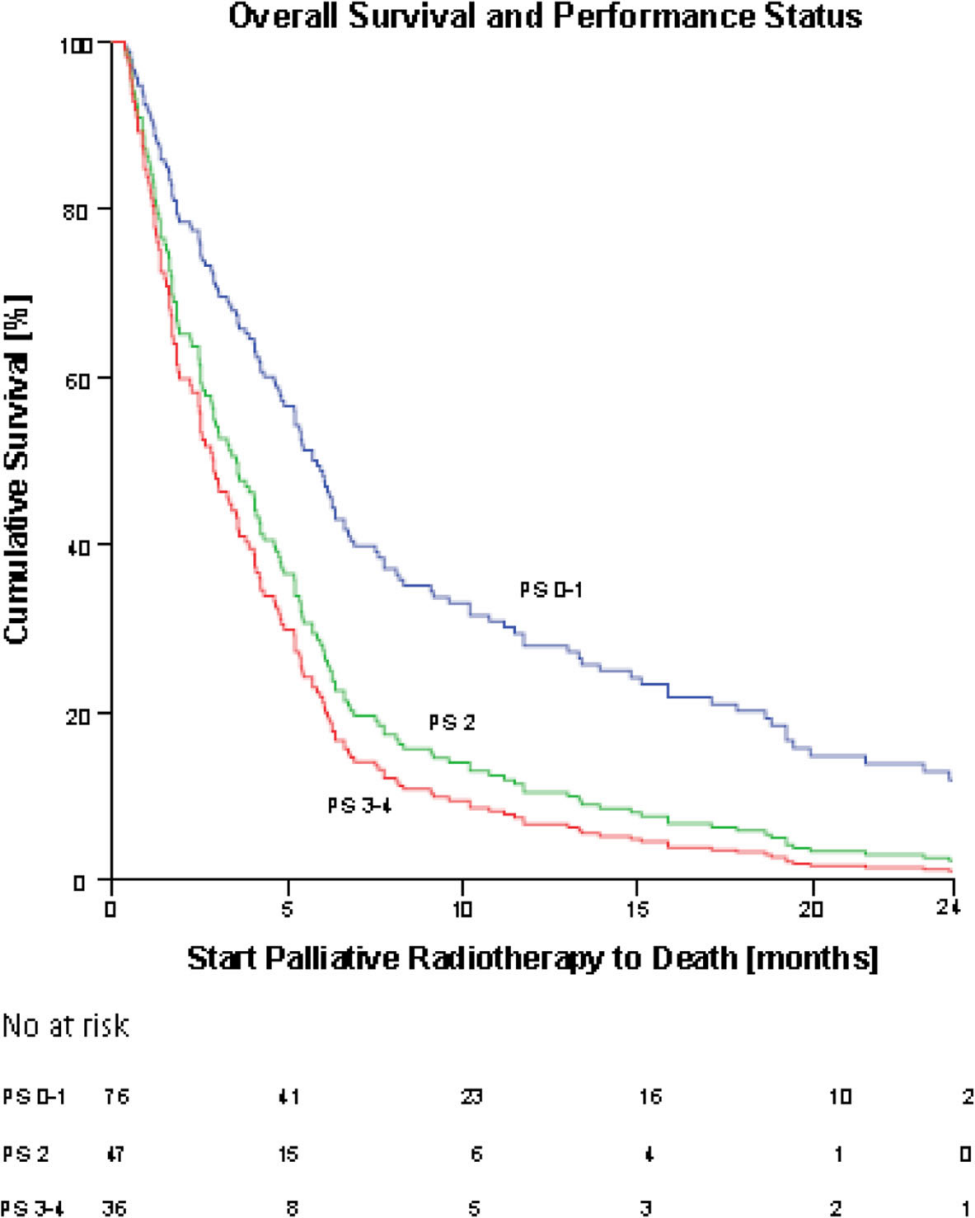
Cox regression analysis showing correlation between OS and PS from prescription of PTR to death. There was a significant difference in OS and PS 0-1 and 2-4. PS 2: hazard ratio (HR) = 1.77, (95% CI: 1.22-2.57), *p* = 0.003. PS 3-4: HR = 2.12 (95% CI: 1.42 to 3.17), *p* < 0.000. There was no statistical difference in OS and PS 2 and 3-4

**Fig. 3:**
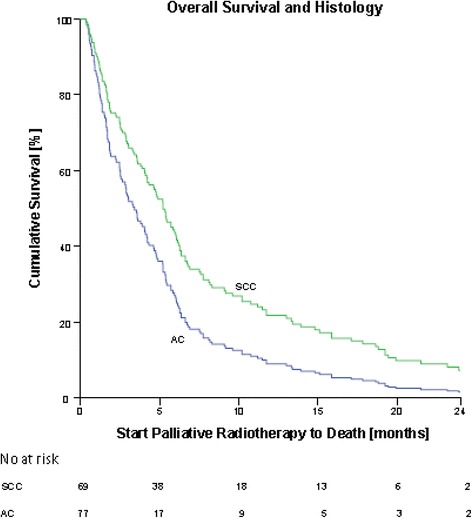
Cox regression analysis showing correlation between OS and histology from prescription of PTR to death. There was a significant difference in OS and histology, favoring squamous-cell carcinoma (SCC) over adenocarcinoma (AC). SCC had a HR = 0.63 (95% CI: 0.46 to 0.89), *p* = 0.007

PS 0 and 1 were grouped in one category as the groups would otherwise be too small for further analysis. Statistical analysis showed a significant difference in OS between PS 0-1, 2 and 3-4 in favor of PS 0-1 (Fig. 2). There was no difference in OS between PS 2 and 3-4. We found a significant difference between OS and histology favoring mixed adeno-squamous-cell carcinoma and squamous-cell carcinoma (SCC) over adenocarcinoma (AC) (Fig. 3). Due to a small number of patients in the category mixed adeno-squamous-cell carcinoma, these patients were excluded for further analysis in the multivariate cox regression analysis (Additional file 1: Figure S1)*.* Stage, age and fractionation schedule had no significant impact on OS (Additional file 2: Figure S2 and Additional file 3: Figure S3)*.*

## Discussion

Our results support our hypothesis that a significant number of patients in our department received futile or insufficient/ineffective fractionated PTR. We estimated that they did not live long enough to achieve the optimal effect of the treatment since 22% died within 30 days of treatment. Furthermore, 5% died before or during treatment. Our data support that PS is the most important prognostic factor. We found a significant difference in OS between PS 0-1 and 2-4 in favor of patients in PS 0-1, but no significant difference between PS 2 and PS 3-4.

A relative small amount of studies have investigated palliative radiotherapy in the last 14, 30 and 60 days of life [15, 21–24]. The heterogeneity among these studies makes a direct comparison with our data difficult. Van Oorschot et al. [24] found that 12.7% of the patients with NSCLC receiving PTR, started treatment less than 30 days before death. This is consistent with our data where 16% started PTR in the last 30 days before death.

We found a median OS of 4.2 months after PTR. This is lower than compared to 4-12 months in other studies [14, 25–29]. This can partly be explained by that 92% in our patient population had stage III/IV disease and a median PS of 2. Almost half of the patients received chemotherapy prior to PTR.

Sundstrøm et al. [30] analyzed data from 301 patients with NSCLC stadium III receiving 3 different fractionated schedules (17Gy/2F, 42Gy/15F or 50Gy/25F) and found that appetite loss, use of steroids and role function loss, but not Karnofsky score, were statistically significant predictors of OS. Gripp et al. [15] looked specifically at patients (all diagnosis) dying within 30 days of palliative radiotherapy to identify prognostic factors and found that Karnofsky score < 50% (WHO PS 3-4), brain metastases and dyspnoea at rest to be independently associated with an unfavourable prognosis. Van Oorshot et al. [24] investigated prognostic factors among 120 patients with NSCLC receiving different fractionated regimens and found that non-metastatic disease and PS, but not comorbidity, were significant predictors for survival. Rades et al. recently found a significant correlation between N and M stage and survival in palliative radiotherapy for locally advanced lung cancer. Karnofsky score > 70 was borderline significant for survival. This was validated in a larger retrospective study [31, 32].

We also found a significant difference in OS and histology showing a better outcome for patients with SCC compared to AC. An explanation could be that AC more often originates in the periphery which gives symptoms later than a central location and is therefore diagnosed in a more advanced stage.

Few studies have looked specifically at histology as a prognostic factor in palliative radiotherapy. In these, no statistical significance has been found [15, 21, 24].

Despite this heterogeneity, none of the above-mentioned studies revealed age as a prognostic factor, as supported by our findings. This raises the question that elderly patients maybe should not be treated different than the younger, as stated by Turner et al. [33] who showed no significant differences in response nor toxicity regarding PTR between two groups of patients >75 or <65 years, respectively.

A differentiation between symptoms and effect of PTR is useful. The rate of palliation is 60-80% for chest pain and haemoptysis while breathlessness and cough are controlled at a somewhat lower rate of 50-70%. General symptoms as fatigue, anorexia and depression are only affected in a minority of treated patients. PTR rarely helps dysphagia and hoarseness [14, 17, 18, 26–29]. At our institution we found that dyspnea and pain were the most frequent indications for PTR. The third most frequent indication was the category “other” which included patients with no clear indication for PTR. But it also included patients with comorbidity that excluded chemotherapy. This is an interesting aspect as two different studies [34, 35] showed that radiotherapy given to asymptomatic patients does not prevent development of disease-related symptoms and has no beneficial impact on QoL or survival. Thus, delaying radiotherapy seems to be acceptable in asymptomatic patients with locally advanced NSCLC.

American Society of Clinical Oncology (ASCO) has published a guide to manage chemotherapeutic agents in terminally ill patients. Chemotherapy provided within the last 14 days of life as well as treatment courses initiated within 30 days of death is considered as overutilization of chemotherapy [36, 37]. No similar comprehensive guidelines exist for radiotherapy. In approximately three-quarters of lung cancer cases, radiotherapy is indicated and most applied radiotherapy is palliative [38]. Since shorter fractionated treatment or high-dose single fraction PTR provides faster symptom relief and causes fewer side effects, [6, 7] this strategy could provide a meaningful alternative compared to a longer fractionated treatment for patients with PS ≥ 2 and hence not be futile or ineffective/insufficient. The number of patients receiving single fraction PTR in this study were too few to conclude this.

## Conclusion

Our study supports the need for guidelines to avoid futile or ineffective/insufficient fractionated PTR at the end of life. The retrospective study design with the moderate number of patients is a limitation to our study. A larger scale prospective study is needed to validate the findings. Following the patients after completion of PTR could supply us with the lacking knowledge of time to effect on symptoms and duration of palliation. An incorporation of QoL by questionnaires should be mandatory in a clinical trial. Also the role of pathological subtypes needs to be validated.

In our study we found that 22% of patients received futile fractionated treatment. PS was a significant prognostic factor for survival. Based on these results, we suggest that PS should be one of the leading factors when choosing fractionated PTR. Patients with PS 0-1 should be considered for fractionated PTR whereas patients with PS ≥ 2 should be considered for high-dose single fraction regime or supportive care alone.

## Additional files

Additional file 1: Figure S1: Cox regression analysis showing correlation between OS and histology from prescription of PTR to death. Mixed AC/SCC is included. There was a significant difference in OS and histology, favoring both SCC and mixed AC/SCC over AC. Mixed AC/SCC had a HR = 0.25 (95% CI: 0.12-0.51), *p* = 0.000. The rest of the results are listed in Fig. 1. (DOCX 26 kb)

Additional file 2: Figure S2. Cox regression analysis showing correlation between OS and age > or <70 years from prescription of PTR to death. There was a trend towards better OS and high age, but this was not statistical significant. Age > 70 years had a HR = 0.79 (95% CI: 0.58-1.09), *p* = 0.15. (DOCX 25 kb)

Additional file 3: Figure S3. Cox regression analysis showing correlation between OS and radiotherapy schedules 25Gy/5F or 30Gy/10F from prescription of PTR to death. There was a trend towards better OS with 30Gy/10F but this was not statistical significant. 30Gy/10F had a HR = 0.74 (95% CI: 0.52-1.04), *p* = 0.08 (DOCX 25 kb)

## Abbreviations

(AC): Adenocarcinoma
(ASCO): American society of clinical oncology
(ASTRO): The American society for radiation oncology
(EGFR): Epidermal growth factor receptor
(NSCLC): Non-small-cell lung cancer
(OS): Overall survival
(PFS): Progression free survival
(PS): Performance status
(PTR): Palliative thoracic radiotherapy
(QoL): Quality of life
(SCC): Squamous-cell carcinoma
(TNM): Tumor, node and metastasis classification of malignant tumors
