# Supplementary figures and images for: Fractionated palliative thoracic radiotherapy in non-small cell lung cancer – futile or worth-while?
Source: BMC Palliat Care. 2018 Jan 5;17:15. doi: 10.1186/s12904-017-0270-4 (PMC5756366; doi:10.1186/s12904-017-0270-4)

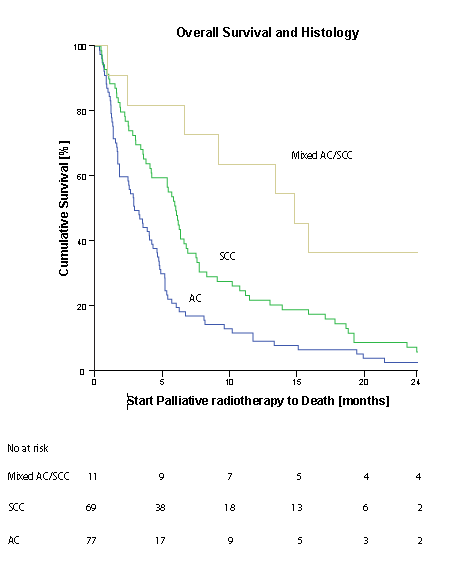

Supplement: Supplementary file 1 — Cox regression analysis showing correlation between OS and histology from prescription of PTR to death. Mixed AC/SCC is included. There was a significant difference in OS and histology, favoring both SCC and mixed AC/SCC over AC. Mixed AC/SCC had a HR = 0.25 (95% CI: 0.12-0.51), p = 0.000. The rest of the results are listed in Fig. 1. (DOCX 26 kb) [file 12904_2017_270_MOESM1_ESM.docx]

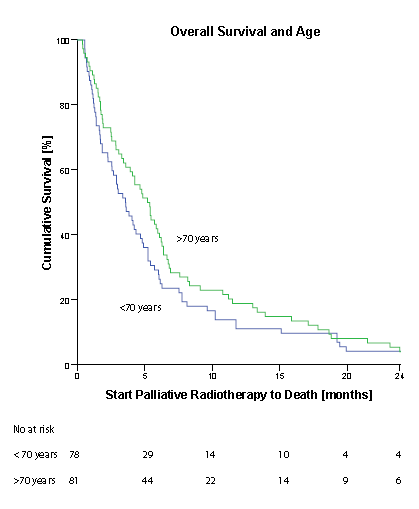

Supplement: Supplementary file 2 — Cox regression analysis showing correlation between OS and age > or <70 years from prescription of PTR to death. There was a trend towards better OS and high age, but this was not statistical significant. Age > 70 years had a HR = 0.79 (95% CI: 0.58-1.09), p = 0.15. (DOCX 25 kb) [file 12904_2017_270_MOESM2_ESM.docx]

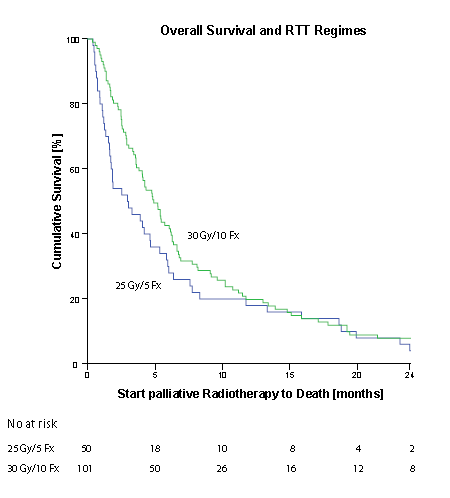

Supplement: Supplementary file 3 — Cox regression analysis showing correlation between OS and radiotherapy schedules 25Gy/5F or 30Gy/10F from prescription of PTR to death. There was a trend towards better OS with 30Gy/10F but this was not statistical significant. 30Gy/10F had a HR = 0.74 (95% CI: 0.52-1.04), p = 0.08 (DOCX 25 kb) [file 12904_2017_270_MOESM3_ESM.docx]
